# Abrogation of Brd4 cellular function contributes to the oncogenic mechanisms of translocation t(15;19)-associated carcinoma

**DOI:** 10.1186/1756-8935-6-S1-P101

**Published:** 2013-03-18

**Authors:** Ranran Wang, Christine M Heifer, Jianxin You

**Affiliations:** 1 Department of Microbiology, University of Pennsylvania, Philadelphia, PA 19104, USA

## Background

Brd4 (bromodomain-containing protein 4) is an important epigenetic reader that plays a crucial role in cellular proliferation and cell cycle progression. Several recent studies have identified Brd4 as a critical therapeutic target in a number of different cancers. Brd4 is also an important target of several oncogenic viruses and the translocation t(15;19) that accounts for a highly aggressive carcinoma. The t(15;19) translocation results in the novel in-frame fusion of the Brd4 N-terminal component with almost the entire sequence of Nut (Nuclear protein in testis) molecule, leading to the formation of a novel fusion oncogene, Brd4-Nut. The mechanism by which the Brd4-Nut fusion product contributes to oncogenesis remains to be clearly defined.

## Materials and methods

In this study, we combined an array of complementary techniques to examine the Brd4 function in chromatin structure maintenance. Using the t(15;19) cancer cell line HCC2429 established from an aggressive, metastatic lung cancer arising in a woman demonstrating a t(15;19), we also performed mechanistic studies to characterize the functional differences between the Brd4-Nut oncoprotein and the native Brd4 isoforms. This study investigated how the Brd4-Nut cancer-predisposing genetic mutation abrogates Brd4 cellular function to contribute to oncogenic progression.

## Results

We applied *in situ* single cell chromatin imaging and micro-coccal nuclease (MNase) assay to show that Brd4 depletion leads to a large scale chromatin unfolding, while a dominant negative inhibitor encoding the double bromodomain (BDI/II) of Brd4 can competitively dissociate endogenous Brd4 from chromatin to trigger severely fragmented chromatin morphology. Using the bimolecular fluorescence complementation (BiFC) technology, we demonstrated that Brd4 molecules interact intermolecularly on chromatin to provide a molecular scaffold for maintaining nucleosomes in a properly organized chromatin conformation. In the t(15;19) HCC2429 cancer cells, Brd4-Nut localizes to discrete nuclear foci and recruits histone acetyltransferases (HATs) to induce histone hyperacetylation, leading to sequestration of Brd4 in these chromatin foci. Using immunofluorescent microscopy and biochemical analysis, we showed that this Brd4-Nut activity significantly enhances Brd4 association with chromatin in HCC2429 cells. Interestingly, exogenous expression of Brd4-Nut in cells without this oncogenic mutation also triggered a tighter Brd4 binding to chromatin. The Brd4-Nut induced Brd4 chromatin sequestration promotes expression of a number of Brd4 downstream target genes, which are important for cell cycle regulation, cellular proliferation and transformation. Brd4-Nut knockdown in HCC2429 cells abrogates HAT recruitment and disperses the histone hyperacetylated chromatin foci, leading to inactivation of Brd4 downstream target genes and inhibition of cellular transformation.

## Conclusions

Our studies demonstrate that Brd4 binding to chromatin is essential for maintaining a properly organized chromatin conformation. We also investigated the structural and functional consequences associated with the t(15;19)-mediated juxtaposition of Brd4 to Nut. These studies revealed additional mechanisms by which this oncogenic mutation perturbs Brd4 function to cause epigenetic reprogramming and malignant progression in the highly aggressive t(15;19) cancers.

